# The Need for a Consensus on the Locution “Central Nuclei” in Striated Muscle Myopathies

**DOI:** 10.3389/fphys.2016.00577

**Published:** 2016-11-23

**Authors:** Anna L. Mazzotti, Dario Coletti

**Affiliations:** ^1^Assointerpreti, Italian Association of Conference InterpretersRome, Italy; ^2^MPS Public SpeakingRome, Italy; ^3^Biology of Adaptation and Aging, CNRS, UMR 8256, INSERM ERL U1164, Institut de Biologie Paris-Seine, Université Pierre et Marie CurieParis, France; ^4^Department of Anatomical, Histological, Forensic Sciences and Orthopedics, Sapienza University of RomeRome, Italy; ^5^Interuniversity Institute of MyologyRome, Italy

**Keywords:** muscle regeneration, myopathy, skeletal muscle histology, central nuclei, centrally located nuclei

“Central nuclei” and “centrally located nuclei” are both widely used expressions to describe the nuclear positioning in skeletal muscle fibers during embryogenesis or muscle regeneration, as opposed to the definitive, subsarcolemmal (i.e., peripheral) nuclear position in adult muscle fibers. The two expressions are mutually exclusive in major databases of scientific literature and authors from different research groups seem to opt for one or the other stochastically, though are consistent with their choice over time. This poses a problem, since a search for one or the other set of keywords retrieves different subsets of articles, limiting the bibliography available. Defining nuclear position is very important in pathology, since many muscle disorders share the mispositioning of nuclei in the muscle fibers (Romero and Bitoun, 2011). In healthy conditions myonuclei are spaced in the periphery of the muscle fibers in such a way that the distance between them is maximized, while they are often found in the center of the myofibers in pathological conditions (recently reviewed by Folker and Baylies, 2013). This phenomenon makes nuclear positioning a common morphological marker for myopathies and the expressions used to describe it are widely used in basic research as well as in diagnosis. Therefore, the issue of using the expression “central” rather than “centrally located nuclei” is not limited to basic research in myology and can affect translational medicine and clinical practice as well, going far beyond a simple matter of semantics. We think there is an urgent need to establish an agreement on the term used in myopathy research as well as in clinical guidelines, “central nuclei” being our favorite choice.

This article means to bring this issue to the attention of the scientific community of myologists, including health care professionals. Below, we discuss in detail the two options and justify our proposal.

## Linguistic considerations

Syntactically both expressions are correct in English. However, “centrally located nuclei” is redundant, since the idea of location is already embedded in the adjective “central.” Indeed, the Merriam Webster Dictionary defines the adjective “central” as: “located in the center of a thing or place / containing or constituting a center / situated at, in, or near the center / centrally placed” and the Oxford Dictionary as: “at the point or in the area that is in the middle of something.” It follows that the only possible usefulness of “centrally located nuclei” may be to make a point; indeed, its use might help stressing the fact that the latter is not the usual localization of nuclei in muscle fibers. On the other hand, “central nuclei” is sufficiently descriptive and more practical, since it is a shorter and simpler expression. This feature, while not representing an argument in favor of “central nuclei” *per se*, can be particularly advantageous when dealing with word-count limits and figure-axis legends, which only allow limited space and privilege short expressions.

If we rule out for “central” the sense of “main or most important,” which is usually inappropriate in scientific results and clinical descriptions of muscle histological features inasmuch as interpretative rather than descriptive, the expression “central” is exhaustive, short, yet unambiguous. Therefore, we conclude that “central nuclei” is the best choice for a topographical description of the muscle nuclei at the fiber center.

## Popularity

Following a search in both Google Scholar and PubMed, “central nuclei” clearly appeared to be more used than “centrally located nuclei” (or its twin expression “centrally positioned nuclei,” which is rare and, therefore, is not taken into account in this article). The search in Google Scholar, extended to full text, was combined with “skeletal muscle” and retrieved about 5600 and 2600 articles for “central nuclei” and “centrally located nuclei,” respectively. A similar 2:1 ratio between the frequency of the two expressions was obtained by a PubMed search extended to title, abstract and keywords. Interestingly, a search by using the Boolean operators “skeletal muscle” AND “central nuclei” OR “centrally located nuclei” produced a number of papers (about 7500 in Google Scholar) close to the sum of the results for each single expression, suggesting that the two locutions are mutually exclusive and are not used in the same context by authors.

Since the search results in the whole literature could be less significant than those in prestigious journals specialized in muscle research or regularly publishing articles on muscle, we conducted a similar search on specific journals and publishing groups (PG), including: Frontiers in Physiology (and the whole Nature PG); Journal of Cachexia, Sarcopenia, Muscle; Skeletal Muscle (and the whole BioMed Central PG); Muscle & Nerve; Neuromuscular Disorders; Neurological Research; Journal of Applied Physiology; Journal of Physiology. In many cases “central nuclei” resulted as the more common expression again, with the noticeable exception of Journal of Cachexia, Sarcopenia, Muscle, as well as Neurological Research, showing that while there is a trend toward using the more popular “central nuclei” a lack of agreement exists even when considering prestigious, international journals written by muscle specialists.

Once more, a roughly 2:1 ratio between the occurrence of “central nuclei” and “centrally located nuclei” was found when searching (by Google search) for the use of the two alternative locutions made by the authors receiving very selective funding by the American Muscle Dystrophy Association or European Telethon associations (supporting research in genetic diseases, and often muscle diseases, in France and Italy). Finally, “central nuclei” was also found in the European Neuromuscular Society guideline on diagnosis and management of limb girdle muscular dystrophies (Norwood et al., 2007), while “centrally located nuclei” was not mentioned in major guidelines or consensus-development conference proceedings (as retrieved in PubMed by using “skeletal muscle,” which gave 52 results).

We think that the fact that “central nuclei” is more widespread should make its fixation in common practice easier, which, in our opinion, would be a beneficial outcome.

## Temporal and geographical distribution

To the best of our knowledge, the oldest example of “central nuclei” present in Google Scholar is in the paper by Godman et al., referring to characteristic nuclei arranged seriatim in the muscle fibers of mice infected by Coxsackie virus (Godman et al., 1952). In PubMed, the first citation containing “central nuclei” in the abstract is the work by Askanas and Engel, describing a human pathologic case in which hypotrophic type I muscle fibers showed central nuclei (Askanas and Engel, 1975). Certainly, someone who contributed to the diffusion of the locution “central nuclei” is Karpati, at the McGill University, with his seminal work on muscle abnormalities and dystrophy (Engel et al., 1968; Karpati et al., 1989).

The expression “centrally located nuclei” appeared later, according to both Google Scholar and PubMed, in the description of regenerating muscle following injury, as in the classical work, by Gutierrez et al., on skeletal muscle regeneration after venom-induced myonecrosis, which paved the way to muscle regeneration studies (Gutiérrez et al., 1984). The oldest Google Scholar citation we found was the work by Kellner and Robertson on experimentally-induced striated muscle necrosis (Kellner and Robertson, 1953).

More recently, users of the “central nuclei” option include renowned researchers in the muscle field in several continents, including North America, Australia, and Europe (McGeachie and Grounds, 1999; McClung et al., 2006; Zampieri et al., 2010a; Pichavant and Pavlath, 2014). Anecdotally speaking, it seems that “centrally located nuclei” is favored by non-English speaker groups, mostly scattered through Europe or East Asia, even though “central nuclei” remains prevalent (Musarò et al., 2007; Coletti et al., 2013; Ikutomo et al., 2014).

Confirming the other observations, the temporal and geographical distribution of “central nuclei” seems wider than that of “centrally located nuclei,” suggesting that more colleagues publishing on muscle are familiar with the first expression and have been so for a longer time.

## Normal and pathological nuclear positioning in striated muscles: examples from the literature

Striated muscles—skeletal and cardiac muscle—differ in terms of the number and position of nuclei. In humans, skeletal muscle fibers are syncytia and their nuclei are peripheral (Allbrook, 1962), while cardiomyocytes are mononucleated (even though they are tetraploid) and this nucleus is central (Adler, 1975; Kikuchi and Poss, 2012). However, some notable exceptions concerning the central positioning of the nucleus can be observed in skeletal muscles as well. For instance healthy Extraocular muscles contain fibers with central nuclei (Carry and Ringel, 1989); this curious feature, together with the abundance of endomysium and the heterogeneous size of the muscle fibers, would be considered a sign of myopathy in other muscles, such as those of the limbs. In addition, the muscle spindles scattered throughout skeletal muscles contain intrafusal fibers, small sensory fibers characterized by central nuclei themselves (Thornell et al., 2015).

Centronuclear myopathies represent a group of inherited diseases in which the majority of muscle fibers have central nuclei. Indeed, chains of centrally located nuclei are the hallmark of this congenital myopathies, proving to be the best example of how central nuclei may represent a histopathological feature of the muscle tissue (Jeannet et al., 2004). In fact, centronuclear myopathies were originally called myotubular myopathies, with a view to highlighting the similarity between pathological myofibers and fetal myotubes (Spiro et al., 1966). Central nuclei have been observed in humans as a marker of myopathy in several striated muscles, and are associated to muscular and non-muscular diseases. Table 1 shows a list of the types of muscles where central nuclei are observed and their underlying pathologies. As shown in this Table, a wide range of skeletal muscles from different regions of the body (neck, trunk, limbs) have central nuclei in their myofibers in pathological conditions. This fact confirms that nuclear positioning is a key feature in the histopathological analysis of skeletal muscle.

**Table 1:** **Examples of striated muscles showing central nuclei in various muscular and non-muscular human diseases**.

**Muscle name**	**Primary disease**	**Bibliografic references**
Medium pharyngeal constrictor	Obstructive sleep apnea	Ferini-Strambi et al., 1998
Deltoid muscle	Dengue	Malheiros et al., 1993
Rectus abdominis	Cancer-associated pre-cachexia	Zampieri et al., 2010a
Biceps brachii	Amyloid myopathy	Manoli et al., 2013
Rectus femoris	Centronuclear myopathy	Hung et al., 1991
Quadriceps	autoimmune Myositis	Zampieri et al., 2010b
Vastus lateralis	Myotonic dystrophy	Andersen et al., 2013

The expressions related to these phenomena are of pivotal importance in basic and applied research as well as in clinical practice.

## Final remarks

In conclusion, we believe a semantic issue exists, having possibly significant consequences on bibliographic searches on topics related to striated muscle regeneration and physio-pathology: that is, authors use two different sets of keywords (“centrally located nuclei” and “central nuclei”) to indicate the same phenomenon, referring to seriatim arranged myonuclei in pathological conditions or during development. However, for the sake of clarity and brevity “central nuclei” is our favorite option now. This expression is the most widely used already and is supported by linguistic considerations. Consequently, its fixation in scientific papers may be desirable, beneficial and conducive to better information retrieval.

## Author contributions

DC conceived the core idea of the manuscript and provided scientific input, while AM provided linguistic counseling. The two authors equally contributed to the writing of the manuscript.

### Conflict of interest statement

The authors declare that the research was conducted in the absence of any commercial or financial relationships that could be construed as a potential conflict of interest. The reviewer ZL and handling Editor declared their shared affiliation, and the handling Editor states that the process nevertheless met the standards of a fair and objective review.
